# Comparison of open and laparo-endoscopic repair techniques for patients with bilateral inguinal hernias

**DOI:** 10.1007/s10029-025-03385-w

**Published:** 2025-06-03

**Authors:** Divyansh Agarwal, Tina Bharani, Nora Fullington, Lauren Ott, Kortney Hodgson, Daelyn McClain, Kaela E. Blake, Michael Reinhorn

**Affiliations:** 1 Department of Surgery, Massachusetts General Hospital, 55 Fruit St., GRB 425, Boston, MA 02114 USA; 2 Department of Surgery, Thomas Jefferson University Hospitals, Philadelphia, PA USA; 3 Boston Hernia, Wellesley, MA USA; 4 Mass General Brigham - Newton Wellesley Hospital, Newton, MA USA; 5 University of Tennessee Graduate School of Medicine, Knoxville, TN USA; 6 Tufts University School of Medicine, Boston, MA USA; 7 20 Walnut Street, Suite 100, Wellesley, MA 02481 USA

**Keywords:** Bilateral inguinal hernia, Open preperitoneal repair, Lichtenstein, Robotic hernia repair, Laparo–endoscopic inguinal hernia repair

## Abstract

**Introduction:**

For primary bilateral inguinal hernias, international guidelines favor a laparoscopic posterior mesh repair due to relatively lower risk of acute and chronic pain, faster recovery, and favorable biomechanical properties compared to open anterior approaches (Lichtenstein, plug and patch, etc.). However, studies comparing open mesh-based bilateral inguinal hernia repairs to bilateral laparoscopic and robotic mesh-based approaches are limited. The Abdominal Core Health Quality Collaborative (ACHQC) registry includes longitudinal data on bilateral inguinal hernia repairs performed via open as well as laparo-endoscopic approaches. We hypothesize that outcomes for bilateral inguinal hernia repair are similar between open and laparo-endoscopic approaches in the ACHQC registry.

**Methods:**

Data from 2012 to 2024 was obtained from the ACHQC registry for individuals who underwent open and laparo-endoscopic bilateral inguinal hernia repair. After adjusting for confounding covariates, 3:1 propensity score-based matching was performed to compare patient-reported quality of life using EuraHS scores between the open, robotic, and laparoscopic bilateral inguinal hernia repair cohorts. Postoperative complications and hernia recurrences were also compared between these cohorts.

**Results:**

In the matched analysis between laparoscopic, robotic, and open repair groups, 575, 524, and 208 individuals, respectively, were included. In the combined analysis comparing laparo-endoscopic to open repairs, data was included for 627 and 211 individuals, respectively, after propensity score matching with 3-year follow up. The open cohort comprised of approximately 40% open preperitoneal and 60% Lichtenstein repairs. The mean age of individuals in this study was 63 years (± standard deviation of 8 years), with nearly 92% of the patients being male (772/838). EuraHS scores up to the 3-year follow-up timepoint did not show statistical or clinical differences between the study cohorts (*p* = 0.19). There were also no significant differences between the rates of hernia recurrence at 3-year follow up, 30-day surgical site occurrences, postoperative bleeding, peripheral nerve injury, venous thromboembolic events, and urinary tract infections between the three cohorts.

**Conclusion:**

For individuals undergoing primary bilateral inguinal hernia repair, all three approaches– laparoscopic, robotic, and open– are comparable in surgical and patient-reported outcomes in the ACHQC registry. Given the high percentage of open preperitoneal repairs in this study, further investigation is warranted to understand if the “open” cohort outcomes are skewed by the combination of both anterior and posterior open repairs in the same group.

## Introduction

The 2018 HerniaSurge International Guidelines recommend a laparo-endoscopic repair for primary bilateral inguinal hernias as it permits repair of both sides through the same incisions and is associated with a faster recovery, lower risk of acute and chronic pain and is cost effective compared to a bilateral open approach [1]. However, this recommendation is based on one non-randomized prospective study [2], and more evidence is needed to confirm or refute this recommendation. Lack of rigorous, high-quality data to assess open mesh-based repairs versus laparo-endoscopic [total extraperitoneal (TEP) and transabdominal preperitoneal (TAPP)] techniques for repair of primary bilateral inguinal hernias has limited the scope of consensus and best practice guidelines on how bilateral hernias should be repaired.

Existing studies have compared TEP versus TAPP [3], and laparoscopic versus open approaches [4, 5] for management of bilateral hernias, finding a relatively low rate of complications, and < 5% risk of conversion to open [6]. Prior work, however, has tended to be underpowered and has found no difference in hernia recurrence risk, postoperative complications and pain [4, 5]. Although it is agreed upon to repair both sides during the same surgical and anesthetic procedure [7], existing comparisons of bilateral inguinal hernia repair (IHR) techniques have yielded conflicting results. While surgeon training and experience affect the repair offered to the patient, how patient-reported postoperative and quality of life (QoL) outcomes differ based on the open versus laparo-endoscopic repair techniques for primary bilateral hernias has remained elusive.

To draw analogy from the primary unilateral IHR literature, while HerniaSurge Guidelines recommend a laparoscopic approach for primary unilateral IHR [1, 8], existing work has demonstrated that open preperitoneal (OPP) repairs can offer similar benefits as laparo-endoscopic approaches. Moreover, open repairs can afford the additional advantages of avoiding general endotracheal anesthesia, contributing to fewer postoperative complications, earlier discharge, reduced pain, and improved patient satisfaction [9]. OPP and Lichtenstein repairs can be performed under IV sedation with local anesthesia. Recent analyses comparing OPP unilateral IHR with laparo-endoscopic (both laparoscopic and robotic) and Lichtenstein repairs [10–12] have demonstrated that open preperitoneal repairs can result in fewer complications, relatively low recurrence risk, and reduced rates of acute and chronic postoperative pain. Additionally, previous work comparing an open mesh-based approach with laparo-endoscopic repairs demonstrated increased financial costs associated with the latter [13, 14], without significant differences in rates of rates of wound complications or hernia recurrence [15].

Based on these observations, we hypothesize that open mesh-based repairs may be a reasonable approach to repair bilateral inguinal hernias at high-volume centers. In this study, we analyzed the Abdominal Core Health Quality Collaborative (ACHQC) database to compare outcomes of patients with primary bilateral hernias who underwent a robotic, laparoscopic, or open repair of both hernias in the same surgery. We hypothesize that outcomes for bilateral inguinal hernia repairs are similar between open and laparo-endoscopic approaches in the ACHQC registry.

## Methods

### Study population

Retrospective data from the ACHQC (https://achqc.org/data*)* registry was collected for individuals who underwent a bilateral hernia repair between August 2012 and January 2024 via an open, laparoscopic, or robotic approach. Patients were excluded who underwent a unilateral inguinal hernia repair, a no-mesh repair, transinguinal posterior approaches, combined inguinal and ventral hernia repair, or repair of recurrent inguinal hernias. Additional exclusion criteria included emergent repairs, contaminated or dirty cases, as well as non-elective cases were excluded. Data collected included patient demographics and comorbidities, surgical details, clinical outcomes, and patient-reported outcomes (PROs) before and after bilateral IHR procedures. This study was approved and deemed exempt by the Institutional Review Board at the Massachusetts General Hospital, Boston, MA (USA).

### Characterization of bilateral IHR techniques

In this study, laparoscopic TAPP and TEP repairs are designated in the “laparoscopic” cohort, and the “robotic” cohort includes the rTAPP approach. The “laparo-endoscopic” cohort includes all of these approaches (laparoscopic TAPP, laparoscopic TEP, robotic TAPP). These laparo-endoscopic techniques have been previously described for unilateral IHRs [10]. Open posterior mesh approaches that do not violate the anterior plane, include OPP, TREPP and Kugel repairs, and are all considered as “OPP” repairs. In this study, all OPP repairs and Lichtenstein (anterior onlay mesh) repairs are included in the “open repair” cohort. Of note, an open preperitoneal repair, as performed by most ACHQC surgeons, has been previously described and published with accompanying steps and atlas images [10, 11]. This study compares outcomes between laparoscopic, robotic and open approaches as well as laparo-endoscopic versus open approaches for bilateral inguinal hernia repair.

### Data collection

Patient-reported QoL and longitudinal clinical outcomes were compared between the patients who underwent bilateral inguinal hernia repair via a laparo-endoscopic (robotic or laparoscopic) or open approach. PROs were analyzed using EuraHS QoL scores at 30 days, 6 months, 1 year, 2 years, and 3 years after surgery. EuraHS is a validated, hernia-specific quality of life assessment tool with scores ranging between 0 and 90, lower scores signifying better QoL [16]. The score is calculated on three domains: pain (range 0–30), restriction of activity (range 0–40), and cosmetic discomfort (range 0–20). EuraHS QoL scores were compared up to a 3-year follow-up period due to significant data attrition (> 80% patient data not reported or lost to follow-up) in the ACHQC beyond that time period. Secondary outcomes include perioperative complications, surgical site occurrence or infection (SSO or SSI), and pragmatic hernia recurrence. Pragmatic hernia recurrence in this study includes clinical recurrence on exam, radiographic recurrence, or a patient-reported bulge using the Hernia Recurrence Inventory (HRI) at the site of either hernia (left or right side) at any timepoint ≥ 1 year after surgery. The HRI is a validated measure of hernia recurrence consisting of three yes-or-no questions regarding whether patients see or feel a bulge, whether they feel that their hernia has recurred, and whether they have pain at their hernia repair site [17]. Less than 20% of the participants had a follow-up of 5 years or more due to patient attrition, so pragmatic hernia recurrence was calculated up to a 5-year period post operatively to maintain adequate statistical power.

Our analytical approach involved two concurrent analyses for patients undergoing bilateral IHR: the first compared laparoscopic versus robotic versus open and the second analysis compared laparo-endoscopic (laparoscopic TAPP, laparoscopic TEP, robotic TAPP) to open (Lichtenstein and OPP) repairs.

### Statistical methods

Patients’ demographics and comorbidities, as well as hernia and operative characteristics were compared between the cohorts. Pearson’s chi-squared and Wilcoxon rank sum tests were used to conduct bivariate tests comparing categorical and continuous covariates, respectively, including comparisons of pragmatic recurrence between the different groups. Using propensity score matching, balanced cohorts were created to minimize effects of systematic differences in baseline covariates. A retrospective power analysis calculation, assuming a false-positive rate of 0.05, demonstrated that after propensity-score matching (PSM), the cohorts maintained at least 68.8% post hoc power to detect a moderate size difference between the different groups. A logistic regression model was used to estimate the propensity score for operative approach conditional on covariates identified a priori. Covariates included in the propensity score model were age, gender, race, BMI, insurance status, ASA class, comorbidities, history of substance use or smoking, history of anti-platelet or anti-coagulation medications, prior pelvic operations or pelvic mesh, and EuraHS quality of life score measured at baseline. A 3:1 nearest-neighbor matching algorithm with a caliper of 0.2 was used to match robotic and laparoscopic repairs with open repairs. Balance was assessed by examining the standardized mean differences of baseline covariates. All comparisons were considered statistically different if *p* < 0.05. Analyses were performed using statistical programming language: *R*, version 4.4.1.

## Results

### Baseline characteristics of patients

Between August 2012 and January 2024, a total of 7,963 individuals underwent bilateral hernia repair in the ACHQC registry. Among the 1,927 patients who met the inclusion criteria, 880 patients underwent a laparoscopic repair (46%), 810 underwent a robotic TAPP repair (42%), and 237 (12%) patients underwent an open repair for bilateral inguinal hernias. The OPP approach was used in 95 patients (40%), whereas a Lichtenstein repair was performed in 142 individuals (60%). After propensity-score matching, all cohorts were balanced with no significant differences between the adjusted groups based on age, gender, race/ethnicity, ASA class, or medical comorbidities (Table 1). Approximately 92% of participants in each group were male, with a median BMI of 26.0, and nearly 30% of the individuals in each cohort are Medicare beneficiaries. Hypertension was the most common comorbidity, present in 30–32% of all participants across groups, and nearly 8% of the participants endorsed active smoking (Table 1). In each group 99% of the patients underwent repair with a permanent synthetic mesh, and ~ 1% of the patients had a biological tissue-derived mesh used. The median follow-up duration was three years (± 2 years).

**Table 1:** Baseline characteristics of individuals who underwent a bilateral hernia repair via a robotic, laparoscopic, or open approach after propensity score matching. Robotic and laparoscopic repairs were grouped as MIS and compared to the open repairs as well. P-values are reported based on Pearson’s chi-squared test. EuraHS scores are reported as mean ± SD

	Laparoscopic	Robotic	Open	*P*-value		Laparo-endoscopic	Open	*P*-value
*N*	**575**	**524**	**208**			**627**	**211**	
Age capped at 90 (mean (SD))	62 ± 8	63 ± 7	62 ± 7	0.91		63 ± 8	63 ± 8	0.46
Gender = Male (%)	533 (93%)	488 (93%)	192 (92%)	0.92		578 (92%)	194 (92%)	0.91
Race/ethnicity = White (%)	531 (92%)	471 (90%)	191 (92%)	0.34		582 (93%)	194 (92%)	0.67
BMI (mean (SD))	26 ± 2	26 ± 2	26 ± 2	0.22		25 ± 3	26 ± 2	0.89
Insurance (%)				0.94				0.42
Private	346 (60%)	306 (58%)	122 (59%)			360 (57%)	125 (59%)	
Medicare	164 (29%)	161 (31%)	64 (31%)			197 (31%)	64 (30%)	
Other/Unknown	65 (11%)	57 (11%)	22 (11%)			70 (11%)	22 (10%)	
ASA class (%)				0.61				0.55
1-2	450 (78%)	406 (78%)	160 (77%)			482 (77%)	163 (77%)	
3-5	125 (22%)	118 (22%)	48 (23%)			145 (23%)	48 (23%)	
Hypertension = Yes (%)	175 (30%)	169 (32%)	66 (32%)	0.8		203 (32%)	66 (31%)	0.77
Diabetes Mellitus = Yes (%)	24 (4%)	23 (4%)	8 (4%)	0.95		30 (5%)	8 (4%)	0.92
Anti-platelet medications = Yes (%)	72 (13%)	57 (11%)	28 (13%)	0.55		81 (13%)	28 (13%)	0.98
Anti-coagulation medications = Yes (%)	22 (4%)	16 (3%)	8 (4%)	0.76		25 (4%)	8 (4%)	0.91
Current Smoker = Yes (%)	40 (7%)	42 (8%)	16 (8%)	0.68		49 (8%)	16 (8%)	0.83
Operative time > 2 h = Yes (%)	82 (14%)	92 (18%)	21 (10%)	0.096		99 (16%)	22 (10%)	0.11
EuraHS overall score at baseline	24 ± 12	24 ± 10	24 ± 13	0.97		23 ± 13	24 ± 10	0.21
EuraHS score for baseline pain	6 ± 4	6 ± 4	7 ± 4	0.95		6 ± 4	7 ± 5	0.4
EuraHS score for baseline esthetics	8 ± 4	8 ± 3	8 ± 3	0.37		8 ± 4	8 ± 3	0.88
EuraHS score for baseline restriction	10 + 9	9 ± 7	10 ± 2	0.84		8 ± 7	10 ± 8	0.14

### Patient-report quality of life

In the matched analysis, there were no significant differences in the postoperative EuraHS QoL scores between the robotic, laparoscopic, and open bilateral IHR cohorts during the 3-year follow-up period (Table 2). When the combined laparo-endoscopic cohort was compared with those who underwent an open repair, the paucity of differences persisted. For instance, at the 3-year timepoint, the combined laparo-endoscopic and open repair cohorts both had a median overall EuraHS score of 0 (*p* = 0.19; Table 2). We additionally performed domain-specific analyses to compare the cohorts in terms of aesthetic, pain, and restriction EuraHS scores. No QoL differences were evident at any time point during the 3-year follow-up in any of the three domains in the individual multivariate (robotic versus laparoscopic versus open) or the combined laparo-endoscopic versus open analyses (*p* > 0.5 for all domain-specific comparisons). These results suggest that the PROs between the three approaches– robotic, laparoscopic, and open– are similar for primary bilateral IHR.

**Table 2:** EuraHS pros compared between individuals who underwent a bilateral inguinal hernia repair via a laparoscopic, robotic, or open technique. Laparoscopic and robotic cohorts were compared individually with each other and the open repair cohort, as well as combined laparoscopic and robotic repairs were grouped as “Laparo-endoscopic” and compared collectively against open bilateral inguinal hernia repairs

Outcome	Laparoscopic	Robotic	Open	*P*-value		Laparo-endoscopic	Open	*P*-value
	(*N*=575)	(*N*=524)	(*N*=208)			(*N*=627)	(*N*=211)	
EuraHS QoL score at baseline survey (score 0-90)	24 (12,39)	24 (10,42)	24 (14,40)	0.97		23 (10,38)	24 (14,40)	0.21
EuraHS QoL score at 30-day survey (score 0-90)	13 (4,29)	14 (4,27)	14 (5,26)	0.73		12 (4,28)	15 (5,26)	0.16
EuraHS QoL score at 6-month survey (score 0-90)	2 (0,6)	1 (0,6)	2 (0,6)	0.54		2 (0,6)	2 (0,6)	0.97
EuraHS QoL score at 1-year survey (score 0-90)	2 (0,5)	1 (0,6)	0 (0,2)	0.11		2 (0,6)	0 (0,2)	0.14
EuraHS QoL score at 2-year survey (score 0-90)	0 (0,4)	0 (0,4)	1 (0,4)	0.81		0 (0,4)	1 (0,4)	0.72
EuraHS QoL score at 3-year survey (score 0-90)	0 (0,6)	1 (0,6)	1 (0,4)	0.32		0 (0,6)	0 (0,3)	0.19

Note: P-value calculated using the Kruskal-Wallis test EuraHS scores are reported as Median (Lower Quartile, Upper Quartile)

### Perioperative outcomes

Further, we examined the differences in post-operative complications among participants undergoing laparo-endoscopic versus open bilateral hernia repair (Table 3). Although not statistically different, 30-day post-operative SSO rates were 3.8% (8/208) for open, 9.5% (55/575) for laparoscopic, and 6.1% (32/524) for robotic (*p* = 0.57), which were largely driven by increased seromas in the laparo-endoscopic groups. The SSO rates between open versus combined laparo-endoscopic groups were also not significantly different (3.8% vs. 8.6%, *p* = 0.08). There were no differences in rates of 30-day surgical site infections requiring procedural intervention, postoperative bleeding, peripheral nerve injury, postoperative respiratory failure, pulmonary embolism, ileus, bowel obstruction, deep vein thrombosis (DVT), or urinary tract infections (UTI) between the cohorts (Table 3).

**Table 3:** Perioperative and 30-day clinical outcomes between the laparoscopic, robotic and open bilateral IHR cohorts after propensity score matching. Laparoscopic and robotic cohorts were compared individually with each other and the open repair cohort, as well as combined laparoscopic and robotic repairs were grouped as “Laparo-endoscopic” and compared collectively against open bilateral inguinal hernia repairs

Outcome	*N*	Laparoscopic	Robotic	Open	*P*-value		*N*	Laparo-endoscopic	Open	*P*-value
		(*N*=575)	(*N*=524)	(*N*=208)				(*N*=627)	(*N*=211)	
30-day readmission: Yes	1307	3	6	0	0.22		838	4	0	0.24
30-day surgical site infection (SSI): Yes	1307	1	0	0	0.53		838	1	0	0.56
30-day surgical site occurrence (SSO): Yes	1307	55	32	8	0.01		838	54	8	0.021
Wound cellulitis: Yes	95	0/55	0/32	1/8	0.004		62	0/54	1/8	–
Wound serous drainage: Yes	95	0/55	1/32	0/8	0.37		62	0/54	0/8	–
Seroma: Yes	95	43/55	23/32	5/8	0.57		62	47/54	5/8	0.078
Hematoma: Yes	95	12/55	6/32	2/8	0.91		62	6/54	2/8	0.27
30-day NSQIP complications: Yes	1307	5	2	0	0.55		838	3	0	0.62
Pain: Yes	38	1/23	1/14	0/1	–		18	0/17	0/1	–
UTI: Yes	38	1/23	0/14	0/1	–		18	0/17	0/1	–
Pneumonia: Yes	38	1/23	0/14	0/1	–		18	1/17	0/1	0.8
Other NSQIP complication: Yes	38	2/23	1/14	0/1	–		18	2/17	0/1	0.31
Operative Time (minutes)	1307				0.001					0.001
0-119		493 (86%)	432 (83%)	187 (90%)				528 (84%)	189 (89%)	
>120		82 (14%)	92 (17%)	21 (10%)				99 (16%)	22 (10%)	
General anesthesia		575 (100)%	524 (100%)	108 (52%)	< 0.0001			627 (100%)	101 (48%)	< 0.0001

Note: Absolute numbers, not percentages, are reported in the table. N is the number of non-missing values. P-value calculated using Pearson’s chi-squared test “Other NSQIP complication” includes complications not captured by ileus, bowel obstruction, PE, DVT, stroke, MI, cardiac arrest, septic shock, renal insuffciciency or failure, peripheral nerve injury, post-operative bleeding, and coma

Intra-operative outcomes were analyzed between groups. The rate of using general anesthesia in the open cohort was half of that used in the laparo-endoscopic cohort [48%, (101/211) vs. 100% (624/624), *p* = 0.01). Rates of pragmatic hernia recurrence were compared between cohorts for patients who developed a hernia recurrence on at least one side after bilateral IHR. At the median 3-year follow-up, recurrences were identified in 13% (13/100) of patients who underwent a laparo-endoscopic repair versus 4% (1/25) of patients who underwent an open repair (*p* = 0.24).

## Discussion

This study compared patient-reported clinical and operative outcomes after primary bilateral IHR between individuals who underwent open, laparoscopic, or robotic repair. No significant differences in patient-reported QoL, hernia recurrences or postoperative complications were identified between the three repair approaches. Although not statistically different, there were lower rates of hernia recurrence (4% versus 13%) and SSOs (3.8 vs. 8.6%) in the open cohort compared to the laparo-endoscopic cohort. These differences could be clinically meaningful, especially at high volume centers. In the open cohort, there were no differences in acute or chronic pain when compared to laparo-endoscopic approaches, similar to a recent study that found comparative outcomes after an open inguinal hernia repair versus laparoscopic or robotic inguinal hernia repair for individuals with unilateral inguinal hernia [15]. Additionally, the open approach demonstrated advantages in terms of shorter operative times and avoidance of general anesthesia.

Previous work has shown that a preperitoneal repair has improved PROs when compared to a Lichtenstein repair in patients with a unilateral inguinal hernia [11, 18]. Multiple RCTs have demonstrated that postoperative outcomes and complications are reduced when doing a TEP when compared to Lichtenstein, with the caveat that there were more intraoperative complications in the laparo-endoscopic route [19, 20]. In this analysis, nearly 40% of the open repairs utilized the OPP approach for bilateral IHR. Since OPP repairs have been shown to have improved outcomes over Lichtenstein, we hypothesize that our findings are driven by the procedure choice of OPP, more than Lichtenstein. OPP has shown similar results to laparo-endoscopic unilateral IHRs while avoiding general anesthesia and a low risk of intra-operative injuries [10]. Our results may have important implications on international practices for bilateral inguinal hernia repairs, as general anesthetic and laparo-endoscopic equipment remain in short supply and are relatively expensive compared to open instrumentation and sedative anesthesia. With studies suggesting lower cost and resource demand associated with open repairs, it becomes imperative to consider this option even in wealthy countries where costly healthcare systems are outpacing their population’s ability to pay for this care. With proper training [21, 22], OPP may be a better alternative to laparo-endoscopic for primary inguinal hernia repair, although this may not represent the general population since the surgeons contributing to the high OPP volume in the ACHQC registry are high-volume inguinal hernia experts located in specialized hernia centers. Additional study is warranted to further investigate this observation.

While this study challenges the developing consensus that a laparo-endoscopic repair is best for bilateral inguinal hernia repair, it is important to understand that the open approaches grouped together in this study are varied with differing approaches to mesh placement and dissection of tissue planes, resulting in the potential for different outcomes. We have recently argued that if “open” IHRs were named according to the anatomical planes that are entered and dissected, we would have four separate categories– open tissue approaches, open anterior mesh approaches, open preperitoneal repairs, and open anterior and posterior mesh approaches [23]. The ACHQC registry breaks “open” repairs into subcategories allowing future analyses to be more nuanced, without having to generalize all open repairs as being equal. Our data analysis was underpowered to perform a direct comparison between OPP and laparo-endoscopic repairs leading to combination of OPP with Lichtenstein repairs.

Limitations of this study include its retrospective nature and the associated attrition in long-term follow-up data. Differences in the surgical training and volume of specific surgeons might bias our results, despite best efforts to account for confounders. While the study design did not control for the influence by a subset of high-volume surgeons, we anticipate that these findings can still apply to experienced centers/surgeons who offer various approaches including laparo-endoscopic, Lichtenstein, and OPP. Nonetheless, this analysis adds to the literature on the clinical utility of an open IHR in patients with primary bilateral inguinal hernias, a theme which has remained underexplored in prior hernia studies.

## Conclusion

Bilateral inguinal hernia repair outcomes in the ACHQC are comparable in terms of quality of life, 30-day complications, and recurrence rates among individuals undergoing primary bilateral inguinal hernia repair, irrespective of the approach– laparoscopic, robotic, or open. Additional research comparing Lichtenstein to open preperitoneal and laparo-endoscopic approaches is needed to elucidate the differences between approaches further. Additional teaching and investigation of OPP should be considered as it is faster, requires less general anesthesia, and may be more cost-effective than laparo-endoscopic repairs, especially in resource-limited settings.
